# Relationship Between Self-Acceptance and Intention to Stay at Work Among Clinical Nurses in China: A Cross-Sectional Online Survey

**DOI:** 10.3389/fpsyt.2022.897157

**Published:** 2022-07-12

**Authors:** Lingling Kong, Fangxia Qin, Aiying Zhou, Shanju Ding, Hua Qu

**Affiliations:** ^1^Department of Psychology, Medical Humanities Research Center, Binzhou Medical University, Yantai, China; ^2^Shandong Mental Health Center, Jinan, China; ^3^Yantai Yuhuangding Hospital, Yantai, China; ^4^Yantai Affiliated Hospital of Binzhou Medical University, Yantai, China

**Keywords:** self-acceptance, intention to stay, clinical nurses, cross-section, psychological nursing

## Abstract

**Background:**

During the pandemic and with the growing shortage of nurses, the problem of how to retain existing nurses was of paramount importance. However, there is limited evidence on the relationship between nurses' self-acceptance and intention to stay.

**Objectives:**

This study aimed to investigate the factors influencing nurses' intention to stay at work, and explore the relationship between self-acceptance and their intention to stay.

**Methods:**

Convenience sampling was conducted to select nurses who worked in a clinical environment during June 2020, in hospitals in Shandong Province, China. Self-designed basic information and two questionnaires, namely, the “self-acceptance questionnaire” and “intention to stay” were adopted. Mean, median, related analysis, and regression analysis were adopted to describe the relationship of self-acceptance and intention to stay on part of Chinese nurses.

**Results:**

A total of 1,015 clinical nurses participated in the survey. The mean score of intention to stay among participants was 22.00. The multiple regression analysis revealed various factors, such as age, family support the work, interest in work, job suitability, type of employment, professional level, weekly working hours, working department and self-acceptance influenced the nurse's intention to stay (β range from −1.506 to 2.249).

**Conclusion:**

Our findings identified several factors that are significantly related to and impact the level of intention to stay among clinical nurses.

## Introduction

There have been 515,192,979 confirmed cases of coronavirus disease 2019 (COVID-19) and 6,254,140 deaths reported worldwide as of 9 May 2022 (1). While in China, 1,336,936 confirmed cases of COVID-19 and 15,450 deaths were reported to WHO (2). Owing to the rapid spread of the virus, the WHO classified the COVID-19 outbreak as a pandemic and as a public health issue on March 11, 2020 (3). The outbreak of a pandemic can have negative psychological outcomes. Due to safety needs and occupational work needs, medical staff including nurses caring for patients are more likely to suffer from negative psychological effects, such as anxiety and depression after stress events or emergencies, as compared to the general population (4–9). The mental and physical health status of clinical nurses can also cause burnout and a wish to leave the job.

In recent years, shortages and high turnover rates among nurses have emerged as a global problem for healthcare managers. According to the State of the World's Nursing Report 2020, it is predicted that without action, the global nurse workforce will fall short by 4.6 million by 2030 (10). By the end of 2019, the total number of clinical nurses was 4.45 million, and there were 3.18 per 1,000 population in China, double the number in 2010 (11). Despite initiatives undertaken in recent years to alleviate the general shortage of nurses, the distribution of nurses is still uneven between urban and rural areas, and between tertiary hospitals and primary care institutions. In addition, with the occurrence of COVID-19, nurses not only have to deal with the health challenges brought by climate change, population aging, and the increasing number of non-communicable diseases but also health challenges such as emerging infectious diseases (12). Strengthening the willingness of nurses to stay in their job is of great practical value for alleviating the shortage of nurses, thereby promoting global health. Research has shown that the intention to stay in the job is an important predictor of retention of nurses (13, 14). Intention to stay in the job refers to an individual's tendency to continue to work with their current employer rather than seek other job opportunities (15). Previous studies have confirmed several factors related to intention to stay or leave, such as organizational characteristics (16–18), job embeddedness (19), job suitability (20–22), and job control (20).

Self-acceptance is individuals' positive attitude toward everything about themselves (23), including their body and moral character. The phenomenon of self-acceptance is generally thought to consist of two factors (24). On one hand, it refers to the ability and willingness to let others see you as you really are. On the other hand, it means being able to evaluate yourself objectively. Self-acceptance is closely related to one's mental health (25, 26). The higher the level of self-acceptance, the greater the degree of mental health. As the primary standard of mental health, nurses with a high degree of self-acceptance can reflect their self-worth and take a more proactive attitude in the process of getting along with doctors and patients, which is conducive to establishing a stable interpersonal relationship, providing better care to patients, and obtaining a greater sense of achievement. This study, therefore, attempts to assess the relationship between self-acceptance and intention to stay among clinical nurses in China.

Self-acceptance can affect the relationship between the nurse and the patient, which in turn, may even affect the patient's recovery. According to our literature review, we could not locate any research about the relationship between self-acceptance and intention to stay among clinical nurses. Thus, we aim to explore the factors affecting the intention to stay and hypothesize a close relationship between self-acceptance and intention to stay.

## Methods

### Study Design, Settings, and Participants

This research, a cross-sectional study, was developed in June 2020 and designed to describe the influencing factors impacting nurses' intention to stay in the job. The convenience sampling method was used to survey participants from hospitals in Shandong Province, China. According to its population structure, social and cultural patterns, Shandong Province is typical of Chinese provinces. Participants invited to complete the research were full-time nurses. This research was conducted using the *Wenjuanxing* tool, which is a commonly used online survey tool in China. Through WeChat (a common social media app), we sent mobile users a web page with the questionnaire (27). Each computer or phone account should just submit one answer.

In this research, all participants were informed that the survey was anonymous and the basic information of participants would not be presented in survey results. They were also informed that this research was voluntary and they could withdraw their participation at any point of time. Informed consent was obtained from the participants before they completed the online questionnaire. However, those who did not provide informed consent were blocked from completing the questionnaire. Data captured by an online survey using the *Wenjuanxing* tool was stored securely and only aggregated data were accessed. The study was approved by Ethics Committee of Binzhou Medical University.

### Instrument

The investigation was conducted on the participants' demographic data, basic information about their work, and two scales. Based on the number of items in the questionnaire, we calculated the sample size to be 10 times the number of items (28). Accordingly, the sample size of this study was (16+6)^*^10 = 220. By adding a non-response rate of 20%, it was decided that the final sample size should be 220 (1+0.2) = 264. Demographic data were collected to ascertain the age, gender, and marital status of participants. The basic information about their work involved questioning participants about the grade of the hospital they worked at, years of employment, educational background, types of employment (29, 30), position, professional level (similar to doctors' academic titles in the USA), and department. This survey also determined the participants' job suitability, which was measured by their perception of whether their character was suitable for the job, interest in their work, and family support. The description of the two scales is as follows.

This survey included general demographic data and two questionnaires. The general data questioned participants about their gender, age, marital status, professional level (the methods to evaluate nurses' professional titles in China are similar to those used to evaluate doctors' academic titles in the USA), department, years of employment, educational background, and type of employment. Employment includes three levels, including *bianzhi*, contract-based, and personnel agent. *Bianzhi* refers to people who have established positions and could be loosely regarded as state administrative staff in China (31). Contract-based nurses do not have a permanent contract, and have fewer work-related benefits than nurses in *bianzhi* employment (29, 30). Personnel agent nurses have a personnel relationship in talent exchange centers. The conditions of their employment are lower than the first two levels, and their work safety is the worse. The survey also asked about the position and experience learning regarding empathy-related courses. The design of the two questionnaires is described below.

### The Self-Acceptance Questionnaire

The self-acceptances questionnaire (SAQ), developed by Cong and Gao, was considered for our research (32). It is commonly used to measure the level of self-acceptance (SA) in China (23, 33). The SAQ has 16 items, divided into two subscales including SA and self-evaluation (SE). The SAQ is a 4-point Likert-type questionnaire, ranging from strongly agreeing to strongly disagreeing with a given proposition. The SA subscale adopted positive scores, while the SE subscale consisted of reverse scores. The total score of the questionnaire ranges from 16 to 64, and the score of the subscale range from 8 to 32. The higher the scores, the higher the degree of SA. In this research, the Cronbach's alpha value of the scales, SAQ, SA, and SE, were 0.843, 0.817, and 0.812, respectively.

### The Intention to Stay

The intention to stay at work was measured by Tao and Wang's intention to stay scale (34). It is a popular scale to measure nurses' probability of remaining in their nursing practice in China (20, 35). This scale has six items, of which items two, three, and six are negative, and the results of which are scored in reverse. The total score ranges from 6 to 30, wherein the higher the total score, the greater is the likelihood of staying in the nursing position. The Cronbach's alpha was 0.778 in this research.

### Data Collection

Using the convenience sampling method, the questionnaires were forwarded to the circle of friends of nurses on WeChat. These questionnaires were released through the *Wenjuaxing* survey tool in June 2020, when the pandemic was still spreading. There were no missing data in this survey.

### Statistical Analysis

Data were analyzed using the SPSS version 25. Descriptive statistics were employed to analyze the scores allocated for age, years worked, the SAQ and the intention to stay scale. Data were expressed as the mean (SD) or median [interquartile range (IQR)], where appropriate. The median was utilized because of the wide range of data on age and years worked. The participants' intention to stay scores of the respondents were compared to their demographic characteristics by performing a *t*-test, and a one-way analysis of variance. Spearman's correlation was employed to explore the relationships among age, years worked, SAQ and intention to stay. Univariate and multiple linear regression analyses were conducted to examine what factors influenced intention to stay. The statistical significance was set at *p* < 0.05.

## Results

### Descriptive Analysis of Participants

In total, 1,015 clinical nurses participated in this survey. As depicted in Table 1, the clinical nurses were primarily female (94.6%) and their median age was 32.00 years. Of all clinical nurses, 76.6% were married, and 72.2% had children. 96.55% of their family support their work. Of all participants, 71.7% reported that they were interested in their work, while 82.4% thought that they had specific characteristic traits to be become a nurse and sustain in this field. Of all participants, 62.7% worked at tertiary hospitals, and 78.8% were university graduates or above. The contract-based employment form accounted for 40.4% of respondent work conditions. Among all participants, 61.5% had a junior professional level, with 88.5% holding the nursing position, while 11.5% holding the head nurse position. The median working time was 9.00 years, 46.3% of their monthly income was below 5,000 RMB; 74.7% worked at ward level, and 94.4% reported working 40 h a week or more.

**Table 1:** Description of the factors of the participants and univariate analysis of Intention to Stay (*n* = 1,015).

**Variable**	***n*** **(%)**	**Mean (SD)**	**T/F**	***p***
Total	1,015 (100)	22.00 (4.74)		
Gender				
Female	960 (94.6)	22.05 (4.73)	1.461	0.144^d^
Male	55 (5.4)	21.09 (4.75)		
Age^a^				
20–29	334 (32.9)	21.53 (4.70)	8.416	0.001^e^
30–39	450 (44.3)	21.78 (4.69)		
40 and above	231 (22.8)	23.10 (4.74)		
Marital status				
Unmarried	225 (22.1)	21.47 (4.77)	2.646	0.071^e^
Married	777 (76.6)	22.11 (4.71)		
Divorced/widowed	13 (1.3)	23.85 (5.43)		
With or without children				
Without children	282 (27.8)	21.48 (4.88)	−2.170	0.030^d^
With children	733 (72.2)	22.20 (4.67)		
Family support the work				
Yes	979 (96.5)	22.14 (4.68)	5.074	0.0001^d^
No	36 (3.5)	18.11 (4.71)		
Interesting				
Yes	728 (71.7)	22.99 (4.50)	11.254	0.0001^d^
No	287 (28.3)	19.48 (4.39)		
Job suitability				
Yes	836 (82.4)	22.74 (4.55)	12.399	0.001^d^
No	179 (17.6)	18.54 (4.02)		
Hospital grade				
Tertiary hospital	636 (62.7)	21.80 (4.80)	−1.711	0.087^d^
Secondary hospital	379 (37.3)	22.33 (4.62)		
Education background				
Professional school	18 (1.8)	21.67 (5.64)	1.011	0.364^e^
3 years college	197 (19.4)	21.58 (4.60)		
University or above	800 (78.8)	22.11 (4.75)		
Employment				
*Bianzhi*	293 (28.9)	22.81 (4.81)	15.179	0.0001^e^
Contract-based	410 (40.4)	21.03 (4.56)		
Personnel agent	312 (30.7)	22.51 (4.69)		
Professional level				
Junior level	624 (61.5)	21.69 (4.65)	6.085	0.002^e^
Middle level	350 (34.5)	22.30 (4.83)		
Senior level	41 (4.0)	24.10 (4.71)		
Position				
Head nurse	117 (11.5)	23.19 (4.66)	2.900	0.004^d^
Nurse	898 (88.5)	21.84 (4.73)		
Years worked^b^				
9 and below	518 (51.0)	21.49 (4.67)	7.049	0.001^e^
10–19	292 (28.8)	22.32 (4.73)		
20 and above	205 (20.2)	22.84 (4.77)		
Monthly income^c^				
Below 5,000 RMB	470 (46.3)	21.53 (4.85)	5.258	0.005^e^
5,000–8,000 RMB	384 (37.8)	22.21 (4.56)		
8,000 RMB above	161 (15.9)	22.84 (4.68)		
Working place				
Outpatient service	257 (25.3)	22.35 (4.54)	1.380	0.168^d^
Ward	758 (74.7)	21.88 (4.80)		
Working time every week				
Below 40 h	57 (5.6)	22.61 (4.47)	9.407	0.001^e^
40 h	499 (49.2)	22.58 (4.56)		
Above 40 h	459 (45.2)	21.29 (4.87)		
Department				
Internal medicine	312 (30.7)	21.18 (4.70)	4.155	0.0001^e^
Surgery	168 (16.6)	22.07 (4.73)		
Obstetrics & Gynecology	31 (3.1)	21.20 (4.57)		
Pediatrics	104 (10.2)	21.66 (4.76)		
Operating room	155 (15.3)	23.28 (4.31)		
ICU	39 (3.8)	21.97 (4.48)		
Else	206 (20.3)	22.50 (4.94)		

a *The median age of the participants is 32.00 (IQR = 11.00) (range:21–57)*.

*Age was categorized according to a previous study (36)*.

b *The median working time of all participants is 9.00 (IQR = 12.00) (range:0–48; 0 means that the individual has worked for <1 year.)*.

*Working time was categorized according to a previous study (27)*.

c *Monthly income was categorized according to a previous study (29)*.

d *p-values are derived from t-test*.

e *p-values were derived from One-way ANOVA*.

### Bivariate Analysis of Intention to Stay

The results of this research have shown that the total scores of the intention to stay of the Chinese clinical nurses during the COVID pandemic was mean = 22.00 (SD = 4.74). All of the 12 research factors, namely, age, children, family support, interest in work, job suitability, type of employment, professional level, working position, years worked, monthly income, working time every week, and working department, were significantly associated with the intention to stay at work (Table 1). Clinical nurses who were older, had children and family support, and job satisfaction were likely to stay longer in their nursing jobs. Nurses who had *bianzhi* employment, worked at a senior professional level, had a head nurse position, more income, longer working hours every week, and worked in the operating room might have a higher level of intention to stay.

### Correlation Analysis of Self-Acceptance and Intention to Stay

Table 2 show cases correlations between SAQ scores, their subscales, and intention to stay. This table depicts that the SA, SE, and SAQ are positively correlated with an intention to stay (*r* = 0.193, *p* < 0.01; *r* = 0.173, *p* < 0.01; *r* = 0.243, *p* < 0.01 respectively).

**Table 2:** Correlation analysis of SA, SE, SAQ, and Intention to Stay (*r^a^*).

	**Mean (SD)**	**Age**	**Years worked**	**SA**	**SE**	**SAQ**
Age	33.36 (7.33)	1				
Years worked	11.61 (8.28)	0.939^**^	1			
SA	21.15 (4.90)	0.113^**^	0.129^**^	1		
SE	19.08 (4.25)	0.103^**^	0.110^**^	0.117^**^	1	
SAQ	40.24 (6.90)	0.145^**^	0.158^**^	0.770^**^	0.680^**^	1
Intention to stay	22.00 (4.73)	0.130^**^	0.130^**^	0.189^**^	0.148^**^	0.235^**^

*SA (Self-acceptance) & SE (Self-evaluation) are subscales of SAQ*.

a *r-values are derived from Spearman correlation analysis*.

** *p < 0.01*.

### Univariate and Multiple Linear Regression Analysis

For the univariate analysis, while age, with or without children, family support the work, interest in work, job suitability, type of employment, professional level, working position, years worked, monthly income, weekly working hours, working department, SA, SE, and SAQ were regarded as the independent variables and intention to stay was the dependent variable. The coding of variables is displayed shown in Table 3. In this analysis, the categorical variables were entered as dummy variables (37, 38). The results revealed that, the scores of intention to stay were associated with all the independent variables (Table 4). The results of the variance factors of intention to stay, the Spearman's correlation analysis and the univariate linear regression analysis (Tables 1, 2, 4) revealed that while the variables of age, with or without children, family support the work, interest in work, job suitability, type of employment, professional level, working position, years worked, monthly income, weekly working hours, working department, SA, SE, and SAQ were employed as independent variables, intention to stay was the dependent variable. The results of multiple linear regression demonstrated that age, family support the work, interest in work, job suitability, type of employment, professional level, weekly working hours, working department, and SAQ were entered in the regression equation (Table 5).

**Table 3:** The assignment of independent variables.

**Variable**	**Assignment**
Age	• Setting 2 dummy variables, • D1:30–39 years = 1, else = 0; • D2:40 years and above = 1, else = 0
With or without children	Without children = 0,With children = 1
Family support the work	No = 0;Yes = 1
Interest in the work	No = 0;Yes = 1
Job suitability	No = 0;Yes = 1
Employment	• Setting 2 dummy variables, • D1:contract-based = 1, else = 0; • D2:personnel agent = 1, else = 0
Professional level	• Setting 2 dummy variables, • D1:middle level = 1, else = 0; • D2:senior level = 1, else = 0
Position	Head nurse = 0; Nurse = 1
Years worked	• Setting 2 dummy variables, • D1:10–19 years = 1, else = 0; • D2:20 years and above = 1, else = 0
Monthly income	• Setting 2 dummy variables, • D1:5,000–8,000 RMB = 1, else = 0; • D2:8,000 RMB and above = 1, else = 0
Weekly working time	• Setting 2 dummy variables, • D1:40 h = 1, else = 0; • D2:above 40 h = 1, else = 0
Department	• Setting 6 dummy variables, • D1:Surgery = 1, else = 0; • D2:Obstetrics & Gynecology = 1, else = 0; • D3:Pediatrics = 1, else = 0; • D4:Operating room = 1, else = 0; • D5:ICU = 1, else = 0; • D6:Else department = 1, else = 0;
SA	Continues variables
SE	Continues variables
SAQ	Continues variables

**Table 4:** Univariate linear regression examining characters associated with intention to stay.

**Variables**	***R*** **^2^**	**β**	**95% Confidence interval**		***P***
			**Lower upper**	**Lower upper**	
Age (ref. 20–29 years)	0.016				
30–39 years		0.252	−0.414	0.919	0.458
40 years and above		1.565	0.776	2.355	0.0001
With children (ref. Without)	0.005	0.719	0.069	1.369	0.030
Family supports the work (ref. No)	0.025	4.030	2.471	5.588	0.0001
Interest in the work (ref. No)	0.111	3.505	2.894	4.116	0.0001
Job suitability (ref. No)	0.114	4.203	3.482	4.924	0.0001
Employment (ref. *Bianzhi*)	0.029				
Contract–based		−1.785	−2.487	−1.084	0.0001
Personnel agent		−0.303	−1.048	0.443	0.426
Professional level (ref. junior level)	0.012				
Middle level		0.605	−0.013	1.222	0.055
Senior level		2.405	0.914	3.896	0.002
Position (ref. Head nurse)	0.008	−1.345	−2.255	−0.435	0.004
Years worked (ref. 9 years and below)	0.014				
10–19 years		0.831	0.154	1.507	0.016
20 years and above		1.359	0.597	2.122	0.0001
Monthly income (ref. 5,000 RMB and below)	0.010				
5,000–8,000 RMB		0.677	0.040	1.314	0.037
8,000 RMB and above		1.311	0.465	2.156	0.002
Weekly working time (ref. below 40 h)	0.018				
40 h		−0.039	−1.328	1.250	0.953
Above 40 h		−1.320	−2.615	−0.025	0.046
Department (ref. Internal medicine)	0.024				
Surgery		0.883	0.001	1.764	0.050
Obstetrics & Gynecology		0.011	−1.723	1.745	0.990
Pediatrics		0.481	−0.562	1.524	0.366
Operating room		2.101	1.196	3.006	0.0001
ICU		0.792	−0.773	2.356	0.321
Else		1.322	0.495	2.149	0.002
SA	0.037	0.188	0.129	0.247	0.0001
SE	0.030	0.193	0.126	0.261	0.0001
SAQ	0.059	0.167	0.125	0.208	0.0001

**Table 5:** Multiple linear regression examining characters associated with intention to stay.

**Independent variables**	**β**	**SE**	**Beta**	***t***	***P***
Age (ref. 20–29 years)					
30–39 years	−0.168	0.456	−0.018	−0.368	0.713
40 years and above	1.929	0.863	0.171	2.234	0.026
With children (ref. Without)	−0.202	0.412	−0.019	−0.491	0.623
Family supports the work (ref. No)	2.144	0.734	0.084	2.920	0.004
Interest in the work (ref. No)	1.683	0.378	0.160	4.450	0.000
Job suitability (ref. No)	2.249	0.446	0.181	5.047	0.000
Employment (ref. *Bianzhi*)					
Contract–based	−1.288	0.442	−0.133	−2.914	0.004
Personnel agent	−0.348	0.451	−0.034	−0.772	0.440
Professional level (ref. junior level)					
Middle level	−0.947	0.43	−0.095	−2.203	0.028
Senior level	−0.036	0.899	−0.002	−0.041	0.968
Position (ref. Head nurse)	−0.410	0.519	−0.028	−0.789	0.430
Years worked (ref. 9 years and below)					
10–19 years	0.766	0.419	0.073	1.827	0.068
20 years and above	−1.563	0.873	−0.133	−1.791	0.074
Monthly income (ref. 5,000 RMB and below)					
5,000–8,000 RMB	0.516	0.342	0.053	1.510	0.131
8,000 RMB and above	0.29	0.498	0.022	0.582	0.561
Weekly working time (ref. below 40 h)					
40 h	−0.147	0.596	−0.016	−0.247	0.805
Above 40 h	−1.506	0.603	−0.158	−2.498	0.013
Department (ref. Internal medicine)					
Surgery	0.461	0.407	0.036	1.132	0.258
Obstetrics & Gynecology	0.075	0.805	0.003	0.093	0.926
Pediatrics	0.494	0.488	0.032	1.013	0.312
Operating room	1.547	0.486	0.118	3.185	0.001
ICU	0.010	0.723	0.000	0.013	0.989
Else	0.346	0.399	0.029	0.867	0.386
SA	0.007	0.045	0.006	0.160	0.873
SAQ	0.101	0.028	0.147	3.552	0.000

*R^2^ = 0.232, Adjusted R^2^ = 0.213*.

## Discussion

An anonymous, convenience sampling method was used to recruit nurses from hospitals in Shandong Province, China. The goal of this research was to survey the level of intention to stay at work among clinical nurses and factors influencing their intention to stay. This study adopted descriptive analysis and gathered basic information about clinical nurses. The study not only integrated basic information but also included the effects of SAQ. Some effects included in this model have rarely been explored in other research studies of clinical nurses.

### Basic Situation of Clinical Nurses' Intention to Stay

SA (Self–acceptance) & SE (Self–evaluation) are subscales of SAQ.

Our findings on intention to stay was higher than previous studies in China (20, 39). By June 2020, a pandemic stage, nurses had more psychological pressure than in normal situations (40, 41); however, nurses' professional skills and care for patients are reflected more. This makes people realize that nurses are not only the executors of doctors' orders but also the first responders to patients' emergencies, which in turn, offers nurses a greater sense of achievement (42, 43), thereby influencing their willingness to stay at work. In addition, with the proposal of the outline of “Healthy China 2030,” the nursing profession has been gained immense presence, and the willingness of nurses to stay in their job has been slightly improved in China.

### Factors Associated With Intention to Stay

The results of the multiple linear regression revealed that the effects of age, family support the work, interest in work, job suitability, type of employment, professional level, weekly working hours, working department, and self-acceptance variables on the intention of Chinese clinical nurses to stay were statistically significant.

Nurses' intention to stay in their job shows an upward trend with age (16), that is to say, the older the nurses are, the more willing they are to stay in the nursing field. One possible reason is that with increasing age, nurses' abilities in all aspects are improved and their professional identity becomes stronger. The results reveal that nurses are more likely to stay in their job if their families support their work, or if they are interested in their job, or if they feel that the job accords with their personality. The family has an important influence on an individual's work and life. Nurses are more willing to stay if their families support their work and have a harmonious and friendly relationship with them (44, 45). Nurses who are interested in their work and feel that their personality matches with the job (job suitability) have greater enthusiasm for work; they can take initiatives to solve problems at work, have a higher sense of professional identity, and a higher level of intention to stay in the nursing practice.

From the results, it can be concluded that the intention of *bianzhi* and contract-based nurses to stay is higher than that of personnel agents (30, 46). In addition, the higher the income of nurses, the stronger their intention to stay. Compared with contract-based nurses and personnel agent nurses, *bianzhi* nurses have more potential for promotion, competitive recruitment, and selection opportunities, and their intention to stay is stronger. Compared with contract-based nurses, the employment conditions of personnel agent nurses is better. Personnel agent nurses and *bianzhi* nurses receive equal pay for equal work, and thus, they are more willing to stay in the nursing practice. There are differences in the intention of nurses to stay with different professional titles (47). Nurses with middle professional positions were less willing to stay in their posts than those with junior professional positions. This may have been linked to their additional work pressure for which they did not receive sufficient remuneration. The greater impact on nurses' intention to stay is the working hours per week; the longer the working hours per week, the greater the clinical workload, and the heavier the workload, coupled with the shortage of nurses, the lower the intention of nurses to stay. The intentions of nurses in different departments to stay were different, with the highest in the operating room. The work of nurses in the operating room has a certain particularity—the working environment is relatively simple, sealed off, and does not occasion contact with the patients' families—these may be important factors governing the operating room nurses' willingness to stay in the job.

This study has shown that there was a positive association between self-acceptance and the intention to stay on part of the Chinese nurses in a large population. During the pandemic period, as the shortage of nursing staff continues to worsen, the problem of how to make the existing nurses continue to engage in their tasks need to be discussed. As an important part of personality and an important influencing factor of mental health (48), self-acceptance plays an essential role in individual growth, life, and work. The results of this survey demonstrated that the higher the level of self-acceptance of nurses, the more likely they were to continue to engage in nursing-related tasks. Nurses with a high score of the SA factor are more likely to adopt a positive coping style in the face of stressful events (49), setbacks, or psychological distress. They have a greater possibility of establishing a good nurse–patient relationship to gain a sense of achievement in their nursing practice, and they would likely be proactive while engaging in nursing tasks. Nurses with high SE factor scores, not affected by misconceptions, may realize that nursing work is an important part of medical practice, and nursing is highly beneficial to the physical and mental recovery of patients. Moreover, such nurses would have a deep understanding of the work they are engaged in and also have a high sense of professional identity.

## Limitations

There are two limitations of this research. On one hand, due to the limitations of manpower and energy, the convenience sampling method adopted in this study is only a survey of data from one of the provinces in China; therefore, the results cannot be generalized for all Chinese nurses. On the other hand, the study results suggest that there is a certain relationship between nurses' SA and their intention to stay, and does not explore whether the intention to stay will relatively be improved by the improvement of the level of SA. Future studies can attempt to explore the changes in nurses' intention to stay after a SA intervention.

## Conclusions

This research revealed the influencing factors of the intention to stay in the job among nurses and the relationship between self-acceptance and the intention to stay. The level of intention to stay was affected by several factors, which include age, family support the work, interest in work, job suitability, type of employment, professional level, weekly working hours, and working department. We also discovered from the results that there was a relationship between self-acceptance and intention to stay among Chinese nurses. Nurses who had a higher level of self-acceptance had a higher level of intention to stay.

## Data Availability Statement

The original contributions presented in the study are included in the article/supplementary material, further inquiries can be directed to the corresponding authors.

## Ethics Statement

The studies involving human participants were reviewed and approved by Ethics Committee of Binzhou Medical University (2019-70). The patients/participants provided their written informed consent to participate in this study.

## Author Contributions

LK, FQ, and HQ contributed to the conception and design of the research. SD, HQ, and AZ collected the data. LK and FQ conducted the data analysis and statistics. LK wrote the manuscript. All authors contributed to the article and approved the submitted version.

## Funding

This study was supported by Humanities and Social Science youth Fund of Ministry of Education of China (Grant No. 18YJCZH070).

## Conflict of Interest

The authors declare that the research was conducted in the absence of any commercial or financial relationships that could be construed as a potential conflict of interest.

## Publisher's Note

All claims expressed in this article are solely those of the authors and do not necessarily represent those of their affiliated organizations, or those of the publisher, the editors and the reviewers. Any product that may be evaluated in this article, or claim that may be made by its manufacturer, is not guaranteed or endorsed by the publisher.
